# Genome-wide methylation is modified by caloric restriction in *Daphnia magna*

**DOI:** 10.1186/s12864-019-5578-4

**Published:** 2019-03-08

**Authors:** Jack Hearn, Marianne Pearson, Mark Blaxter, Philip J. Wilson, Tom J. Little

**Affiliations:** 1 Department of Vector Biology, Liverpool School of Tropical Medicine, Liverpool, UK; 2 Institute of Evolutionary Biology, School of Biological Sciences, University of Edinburgh, Edinburgh, UK; 3 School of Energy, Geoscience, Infrastructure & Environment, Heriot-Watt University, Edinburgh, UK

**Keywords:** Epigenetics, *Daphnia*, DNA methylation, Caloric restriction, Nutrition, Bisulphite sequencing, Differential methylation, Genomics

## Abstract

**Background:**

The degradation of epigenetic control with age is associated with progressive diseases of ageing, including cancers, immunodeficiency and diabetes. Reduced caloric intake slows the effects of ageing and age-related disease in vertebrates and invertebrates, a process potentially mediated by the impact of caloric restriction on epigenetic factors such as DNA methylation. We used whole genome bisulphite sequencing to study how DNA methylation patterns change with diet in a small invertebrate, the crustacean *Daphnia magna*. *Daphnia* show the classic response of longer life under caloric restriction (CR), and they reproduce clonally, which permits the study of epigenetic changes in the absence of genetic variation.

**Results:**

Global cytosine followed by guanine (CpG) methylation was 0.7–0.9%, and there was no difference in overall methylation levels between normal and calorie restricted replicates. However, 333 differentially methylated regions (DMRs) were evident between the normally fed and CR replicates post-filtering. Of these 65% were hypomethylated in the CR group, and 35% were hypermethylated in the CR group.

**Conclusions:**

Our results demonstrate an effect of CR on the genome-wide methylation profile. This adds to a growing body of research in *Daphnia magna* that demonstrate an epigenomic response to environmental stimuli. Specifically, gene Ontology (GO) term enrichment of genes associated with hyper and hypo-methylated DMRs showed significant enrichment for methylation and acyl-CoA dehydrogenase activity, which are linked to current understanding of their roles in CR in invertebrate model organisms.

**Electronic supplementary material:**

The online version of this article (10.1186/s12864-019-5578-4) contains supplementary material, which is available to authorized users.

## Background

Epigenetic modifications play a key role in patterning gene expression and organismal development. This is particularly evident when epigenetic control degrades, resulting in progressive diseases in humans, including cancers, immunodeficiency and diabetes [1]. The degradation of epigenetic control with age is proposed to occur in a drift-like process. One mechanism that may rescue age-related epigenetic dysregulation is caloric restriction (CR) – reduced caloric intake without malnutrition or loss of micro-nutrients. CR slows the effects of aging and postpones the development of age-related diseases [2–4]. Extension of lifespan is observed in invertebrates and vertebrates, including in yeast, spiders, copepods, worms, silkworms, fish, non-human primates and *Daphnia* [4–10]. The marine copepod crustacean *Paracartia grani* is an example of this [7], and the observed lifespan increase is coupled to lower cumulative egg production in comparison with cohorts fed a high-food diet. This is interpreted as an evolutionary trade-off that allows survival during food shortages that increases the probability of recruitment for some offspring [7, 11]. In rhesus monkeys and mice, a caloric restriction of 30 and 40% respectively appears to reduce epigenetic drift in methylation and increases lifespan. In rodents lifespan can be extended by up to 50% [12]. CR may also delay onset of a spectrum of diseases including cancer, kidney disease, autoimmune disease and diabetes [13–15], as well as neurodegenerative diseases [16, 17].

Mammals have been the focus of studies linking methylation, CR, and ageing due to the high rates of methylation in vertebrates, and the regulatory effect of methylation on disease progression in humans. Conversely, the lower genome-wide rates of methylation in arthropods [18] has hindered study of methylation-derived responses to the environment in this group, but this is now changing [8, 18–25]. Especially as the model organism *Drosophila melanogaster,* along with other surveyed Dipterans, exhibits negligible CpG methylation [18].

### Genome-wide percentage of DNA methylation is low in arthropods

DNA methylation, a reversible covalent modification that regulates gene expression, is the best-studied epigenetic mechanism. DNA methylation of cytosines occurs when DNA methyltransferase enzymes (DNMTs) transfer a methyl group onto cytosine [26] to create a 5-methylcytosine. This can occur at a cytosine immediately followed by guanine (CpG site), or as occurs in plants at a CHH or CHG site; where H can be any of A, T or C. In mammals around 70% of CpGs are methylated [27], whereas the genome-wide rate of CpG methylation in invertebrate species sampled to date is much lower, from 0% in *Drosophila* to 15% in the oyster *Crassostrea gigas* [18, 28]. The crustaceans *Daphnia magna* and *Daphnia pulex* (Arthropoda: Crustacea) have genomic CpG methylation of 0.52–.74 and 0.7% respectively [25, 29].

In mammals, certain insect orders [18], and the crustacean *Daphnia*, there are three DNMT enzymes. The gene products of DNMT3a and DNMT3b establish methylation de novo, while DNMT1 maintains methylation, and DNMT2 has no known role in DNA methylation. DNMT3 has been lost the most among insects and is present in only four orders [18]. Despite the apparent key role of this gene, the genome of the silkworm (*Bombyx mori*) which lacks DNMT3 along with other *Lepidoptera* shows evidence for DNA methylation [8, 18].

CpG methylation can increase or decrease gene expression dependent on the location of the methylation. In mammalian promoter regions, which can be rich in CpGs and are known as CpG islands, it represses expression of the gene. Further to this, many mammalian CpG islands are also enriched for permissive chromatin modification, which condenses the structure of chromatin and further prevents transcription. In contrast, methylation of gene bodies in mammals leads to an increase in expression of the effected gene. Invertebrates have few CpG Islands, and methylation predominantly occurs in gene bodies, and is enriched in exonic sequence to form a mosaic-like pattern genome-wide [30–33]. *Daphnia magna* is no exception to this [25, 29]. The role of gene-body methylation in invertebrates is not as clear as in mammals, but it is implicated in facilitating stable transcription of housekeeping genes and mediating alternative splicing [33–35]. This link to alternative splicing has been identified in *D. magna* [22, 25]. In *Daphnia magna* and *pulex* there is a also positive correlation between highly methylated genes and gene expression in the germline [25]. More specifically, methylation of exons 2–4 is significantly associated with this effect, as it is in humans [25, 36]. In honeybees, differential methylation of CpGs is potentially associated with differential gene expression through ‘priming’ of genes in response to intruders [20]. Interestingly, in silkworms there is no correlation between methylation in promoters and gene expression [8], suggesting invertebrates and vertebrates differ in their usage of CpG methylation.

Identifying differentially methylated regions in organisms with low-levels of methylation, like most arthropods, lacks a standardised approach at present, but DMRs are detectable as a growing body of research shows. This may reflect the nature of invertebrate methylation, with clusters of methylated CpGs interspersed by long, fallow, regions. In the above honeybee study, the authors identified differentially methylated regions between the brains of aggressive and control bees [20]. In *Daphnia*, transgenerational inheritance of CpG modifications has been shown in response to salinity, changes in which impact life-history traits, toxic cyanobacteria, and radioactivity via a whole genome bisulphite sequencing approach [22–24].

### Caloric restriction and DNA methylation

The relationship between diet and CpG methylation, and subsequent impact on ageing and health, is well established in mammals. Indeed, DNA methylation may be a predictor of biological age [37, 38], and a reduction in expression levels of DNMT enzymes leading to a global loss of genomic methylation is associated with ageing humans [39]. Although promoter regions of certain genes become hypermethylated with age [40]. This is in contrast to honey bees in which an increase in de novo methylation activity through DNMT3 is observed [19]. Inhibition of DNMT activity increased lifespans of treated bees, but in a CR independent manner [19]. Specific examples of a diet by methylation interaction include the expression of DNMTs which have elevated expression in response to CR in cancer cells, counteracting the global hypomethylation [41] observed during ageing. CR also causes a reduction in lipid metabolism gene expression by DNA methylation of gene bodies in mouse livers [42]. As a result, older mice undergoing CR were protected from fatty degeneration, visceral fat accumulation, and hepatic insulin resistance compared to controls. In rats and monkeys short-term CR in older individuals ameliorates the effects of ageing with respect to disease markers, oxidative stress and damage, and increases the expression of longevity related genes [43, 44].

### Caloric restriction and CpG methylation in *Daphnia*

Our work aims to determine if an experimentally controlled nutritional environment directs changes in methylation status in the crustacean *Daphnia magna*. Species of Daphnia are well characterised for their response to CR [45], including clear maternal effect phenotypes in *D. magna* [9, 10, 46–48]. However, increased lifespan is not a universal response among *D. magna* strains (T. Little, unpublished data) which can exhibit inter-clonal differences in lifespan and other age-related parameters such as age and size at first reproduction when sampled from the same habitat [49]. This is also true for *D. pulex* where increased lifespans are observed under CR [50–52] but not always [53, 54]. We performed whole genome bisulphite sequencing of CR and normally-fed (NF) replicates of *D. magna* of the the same *Kaimes* strain as prior studies demonstrating a maternal effect response to CR. We identify regions of differential methylation using an approach that successful in previous studies focused on differential modifications at CpG in response to environment-derived treatments [22–25] including salinity and exposure to the toxic cyanobacterium *Microcystis aureginosa*. *D. magna* show the classic response of longer life under CR and strong maternal effects; the offspring of calorie-restricted mothers being larger and more resistant to pathogens than their counterparts from better fed mothers. Provisioning of offspring, e.g. with carbohydrates, protein or fats, is one explanation for these maternal-effect phenotypes, and epigenetic processes, such as methylation, are also potentially key regulators in these plastic responses to fluctuating environments.

*Daphnia* have many attributes that make them favourable for epigenetic study. It has long been known that they undergo adult tissue regeneration unlike other invertebrates, akin to humans [54–56]. Furthermore, they reproduce clonally, which permits the study of epigenetic changes in the absence of genetic variation. Clonal replicates are equivalent to identical twin studies, but with an experimentally chosen number of -uplets. CpG-based methylation occurs in *Daphnia*, its genome encodes all three DNMT enzymes orthologous to mammalian DNMT enzymes, and a growing body of research is showing that global methylation patterns in *Daphnia* change in response to a variety of environmental factors [21–25, 29, 57, 58].

## Results

### Methylated sites prediction

**Fig. 1:**
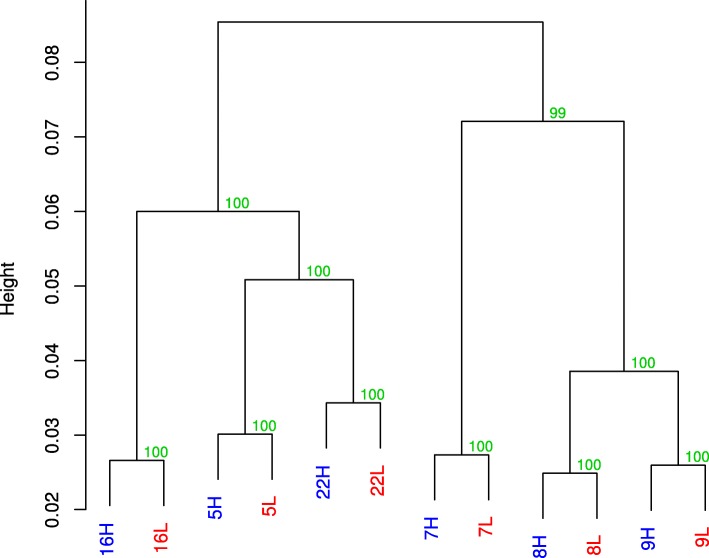
Dendrogram showing replicates cluster by mother and not by treatment in global CpG similarity. Mother is indicated by number, blue H: normal food and red L: calorie restricted replicate. Node support values in green were assessed by 10,000 bootstraps in pvclust, bootstrap probability and approximately unbiased methods had identical values. Dendrogram created using ward.D2 method in methylKit and pvclust. Number refers to mother from which that replicate was derived; H for normal food diet, L for caloric restriction

We performed whole genome bisulphite sequencing (WGBS) of calorie-restricted (CR) and normally-fed (NF) *D. magna* to generate data for identification of methylated C residues and exploration of the C methylation response to CR. Trimmed bisulphite-converted reads aligned to the *D. magna* strain 32 converted genome [48] using Bismark [59] exhibited lower mapping efficiencies than standard short-read alignments, as is typical of WGBS [60], with 20–32% of reads not aligning to the reference genome (Additional files 1 and 2). Of the aligned reads, 29–38% of reads were discarded as PCR duplicates, and 6–10% of the remainder contained predicted CHH or CHG methylated sites. These CHH and CHG site containing reads were removed from analyses, as *D. magna* contains negligible CHH/G methylation [29] meaning they are indicative of bisulphite unconverted reads. This resulted in average replicate read coverages of 8- to 12-fold. Global CpG methylation was 0.7–0.9% in all samples, and no difference in overall methylation levels was observed between NF and CR replicates paired t-test two-tailed *p*-value = 0.17). Removal of polymorphic CpG sites using variants predicted from the bisulphite-unconverted data had little effect on the total number of sites (Additional file 1). This was necessary as at a polymorphic C/T site the T allele can be miscalled as an unmethylated C causing an underestimation of the methylation profile at that site. After filtering, 99.2% of sites were retained per replicate, with average total sites across replicates going from 6.9 million to 6.85 million. Hierarchical clustering of replicates by methylation status demonstrated that mother had a stronger effect on global methylation status than nutritional treatment (Fig. 1). Thus, it was necessary to incorporate pairing information into our statistical test. We did this by performing paired t-tests of CpG sites in the Bioconductor R package “Bsseq” [61].

### Differential methylation assessment

**Fig. 2:**
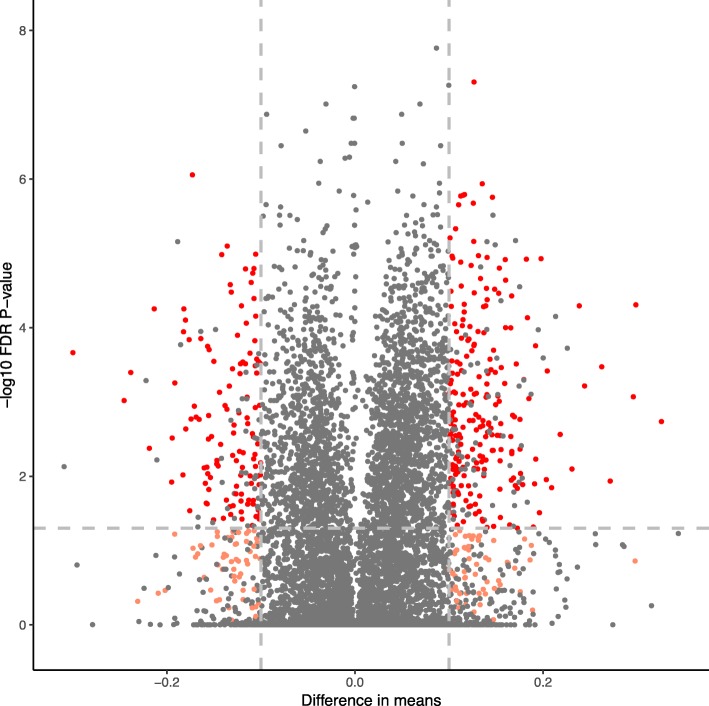
Volcano plot of per-region FDR corrected *p*-values against difference in mean methylation between caloric restricted and normal food conditions. Red dots are DMRs that passed t-statistic filtering and had a region *p*-value below < 0.05, these were retained for further analysis. Pink dots are DMRs that passed t-statistic filtering but were not significant by corrected *p*-value. Black dots are none-significant DMRs by the chosen criteria, dashed grey vertical lines indicate the difference in means (0.1, − 0.1) required to accept a DMR along with a t-statistic of − 4.6 and 4.6 and 3 CpG sites, black dots with a *p*-value less than 0.05 and greater than [0.1] difference in means failed one of these two conditions. Horizontal grey dashed line indicates a *p*-value threshold of 0.05

After filtering for coverage, 4.15 million CpG sites were retained for differential methylation testing. Bsseq testing of differential methylation revealed 8764 potential regions using t-statistic cut-offs of − 4.6, 4.6. This was filtered to 453 DMRs with at least three methylated CpGs and a mean difference greater than 0.1. These DMRS were further filtered after combining raw per region *p*-values into one *p*-value (false discovery rate correction < 0.05) in *comb-p* [62]. This resulted in 333 regions (Fig. 2. Volcano plot of FDR corrected *p*-values) adapting Herb et al. [20]. Of these 117 (35%) were hypermethylated in the CR group versus normal food, and 216 (65%) were hypomethylated in the CR group versus normal food. Only a fraction of these DMRs exhibited a mean difference of greater than 20% in either direction at 4% (5/117) for hyper- and 6% (12/216) for hypomethylated regions respectively. DMRs were 161 base-pairs (bp) and 193 bp long on average for CR hyper- and CR hypo-methylated regions, respectively and ranged from 18 to 602 bp in length. There were from three to 20 CpGs per cluster with an average of six. CR hypermethylated DMRs overlapped 165 gene predictions in the *D. magna* gene set, while CR hypomethylated DMRs overlapped 284. The majority of these overlapped exonic sequences for both hypo- and hypermethylated regions: 82% (234/384) for CR hypomethylated DMRs and 81% (133/165) for CR hypermethylated DMRs. Only 33 DMRs (10%) did not overlap a predicted gene body. The increase in number of DMR containing genes versus total DMRs reflects overlapping or redundant predictions in the *D. magna* genome annotation version 2.4 [63]. Because of this we used a non-redundant set of genes from the *D. magna* gene set as input to GO term enrichment, which resulted in 192 hypo- and 115 hypermethylated genes being considered for functional enrichment. Annotations for these genes are given in Additional files 3 and 4, a greater portion of genes are uncharacterised for hypo- than hypermethylated genes at 28 to 10% respectively. Two genes contain DMRs in both directions (Table 1), one encodes for a latent nuclear antigen and the other a heat shock cognate (Hsc-70) interacting protein. For both genes the exon (exon 2) closer to the predicted start of the gene is hypomethylated, and the 3′ exon is hypermethylated (exon 4 and 6).

**Table 1:** Differentially methylated regions in opposite directions overlapping the same genes

Gene	Genomic Position	DMR	Length of DMR	Condition in CR	Number of sites	Corrected *P*-value	Exon	Strand
Dapma7bEVm000710	scaffold02569: 76199–79,126	76,729–77,106	377	hypo	16	0.0007575	2 of 8	+
Dapma7bEVm000710	scaffold02569: 76199–79,126	77,694–77,926	232	hyper	5	0.001717	6 of 8	+
Dapma7bEVm003762	scaffold01005: 527546–535,133	534,741–534,857	116	hypo	9	0.009821	2 of 6	–
Dapma7bEVm003762	scaffold01005: 527546–535,133	534,106–534,213	106	hyper	4	0.006839	4 of 6	–

Gene: gene name, Genomic Position: gene coordinates, DMR: coordinates of the differentially methylated region, Length of DMR: length in bp, Number of site: number of methylated CpGs in region, Corrected *P*-value: FDR corrected *p*-value for that DMR from comb-p analysis, Exon: which exon of the gene DMR occurs in followed by number of exons for that gene, Strand: which genomic strand the gene occurs on

### GO term enrichment in methylated genes and DMRs

GO term enrichment was explored using the “weight01” algorithm in topGO (downloaded from: https://bioconductor.org/packages/release/bioc/html/topGO.html) and molecular function (MF) and biological process (BP) terms are reported. The enrichment analysis showed significant over representation in one molecular function “acyl-CoA dehydrogenase activity” (GO:0003995) GO term and one biological process “methylation” (GO:0032259) GO term for genes associated with the DMRs (Table 2, Additional file 5), both of which were hypomethylated under CR in all categories of gene tested (all, > 1 CpG, > 3 CpGs). This is not altogether surprising as the number of gene containing methylated CpGs was a high fraction of the total annotated genes (75% for > 1 CpG, and 68% for > 3 CpGs) and is keeping with previous observations [25]. There were two genes associated with acyl-CoA dehydrogenase activity and three with methylation, none of which overlap between terms. The methylation genes consist of two ribosomal RNA methyltransferases and a Cap-specific mRNA (Nucleoside-2′-O-)-methyltransferase, none of which are a member of the DNMT gene family. Both of the acyl-CoA dehydrogenase genes are medium-chain specific acyl-CoA dehydrogenases.

**Table 2:** Gene ontology functional enrichment of DMRs

GO ID	GO term	Ancestors	Direction	CpG 0	CpG 3	All
Biological process						
GO:0032259	methylation	2	Hypo	Yes	Yes	Yes
Molecular function						
GO:0003995	acyl-CoA dehydrogenase activity	4	Hypo	Yes	Yes	Yes

Table lists all significant terms identified in the Biological Process and Molecular Function categories with the ‘weight01’ algorithm, which accounts for GO term hierarchy. Genes of interest were tested against all genes, genes containing at least one methylated CpG and genes containing at least 3 methylated CpGs as per the DMR filtering of greater than 0 and 3 CpGs

## Discussion

### Global methylation and differentially methylated regions

The global *D. magna* CpG methylome of ~ 0.7%, consistent across replicates, is similar to that reported previously for different clones of *D. magna* (0.74 and 0.5% respectively) [25, 29]. Slight differences between studies may be related to *D. magna* strain differences, or differences in different data filtering methods [29]. In line with previously observed methylation patterns in *Daphnia* [22–25] and arthropods [30, 32, 64] in general, the majority of DMRs were identified in exonic regions of gene bodies. The magnitude of change in methylation of DMRs is mainly between 10 and 20% (Fig. 2), with some outliers. Indeed, the greatest difference between treatments was only 33% for a DMR hypomethylated under CR overlapping both exonic and intronic sequence in 40S ribosomal protein S26 (Additional files 3 and 4, genes with exons overlapping DMRs of greater than 20% mean difference). This could reflect our whole organism-based study in which large changes in specific tissues are moderated by no change the majority of tissues for that genomic region. These differences in mean methylation are in line with that for *D. magna* response to *Microcystis* stress, where differences up to 40% were observed [22]. However, the comparison is not direct as that study compared difference between methylation at gene bodies rather than DMRs. The extent to which changes in magnitude of DMRs or gene bodies is functionally relevant is currently unknown in *Daphnia*, and arthropods in general. It is possible that the low, but significant, differences in mean methylation > 10% have little functional effect, and as such requires validation through future studies. In *Daphnia* and other arthropods there is a correlation between DMRs and alternative splicing [22, 24, 25, 30], and is proposed to form an adaptive response to environmental pressures [22, 24, 25]. Increased gene expression is associated with high-levels of germline methylation in *Daphnia* [25], but the impact of environmentally-induced changes on gene expression is not yet understood beyond alternative splicing [22].

There are two genes that contain DMRs methylated in different directions (Table 1), in both cases the first exon (exon two in both genes), has the greater number of methylated CpGs versus the later exon. This is consistent with Kvist et al. [25] as exons two and three in *D. magna* are the most methylated exons genome-wide, with a decline in methylation in subsequent exons. It remains for future work to dissect if the differential methylation in these genes results in any change in gene expression or variation in alternative splicing at these loci.

### Enriched GO terms hypomethylated under CR

The Biological Process GO term ‘methylation’ was enriched in or DMRs and associated with two ribosomal RNA methyltransferase genes and a Cap-specific mRNA (Nucleoside-2′-O-)-methyltransferase gene. The biological roles of these genes are poorly understood, but one hypothesis is that RNA methyltransferases are regulators of global protein translation [65]. One rRNA methyltransferase, NSUN5, is part of a conserved mechanism that modulates ageing [66], as reduced levels of this gene increases lifespan and stress resistance in yeast, worms and flies [66]. Another mechanism by which RNA methyltransferases may act is in protein synthesis that determine cell size, which is hypothesised to explain the small stature of organisms lacking rRNA methyltransferases [65]. This is concordant with the observed phenotype of adult CR *Daphnia* which are smaller than NF individuals, although functional validation would be required to link RNA methyltransferases causally to size difference phenotypes in *Daphnia*. Cap-specific mRNA (Nucleoside-2′-O-)-methyltransferase adds a methyl group to at the ribose 2′-O position of the first transcribed nucleotide, which is important for discriminating self RNAs from foreign RNAs produced by viruses [67, 68]. There is no clear link to caloric restriction with this gene.

The enriched molecular function GO term, Acyl CoA dehydrogenase activity, is associated with two medium-chain specific acyl-CoA dehydrogenase genes. These genes are involved in the breakdown of fatty acid molecules through β-oxidation. Reduced activity of this class of genes in humans impairs the β-oxidation pathway which leads to an intolerance to fasting [69]. There are no studies linking these genes to diet in worms or flies as yet, however they are implicated in heat adaptation through the stabilization of lipid membranes [70]. Broadly interpreted, lipid metabolism is strongly linked to caloric restriction-derived lifespan increase in flies [71], but the specific role of acyl CoA dehydrogenases remains to be elucidated. DNA methylation may act in concert with other mechanisms in response to CR as has been shown with miRNAs [48], but most-likely also through histone modifications [72]. A focus for future research will be the interaction of these different mechanism and their impact, or lack thereof, on global gene expression changes that result in the observed *Daphnia* CR phenotypes.

This experiment was performed with clonally reproducing *Daphnia*. This has the large advantage of a genetically uniform background on which to base results [72], but caution is required when extrapolating our results to sexually reproducing organisms. As discussed by Kvist et al. [24], it is not known if differential methylation is maintained through both parthenogenesis (mitotic) and sexual reproduction (meiosis) as only parthenogenetically transmitted phenotypic changes have been studied. This is also the case for maternal effects resulting from CR in the strain of *D. magna* used here [9, 10, 46, 48]. Jeremias et al. [23] speculate that parthenogenetic species may use epigenetic modifications to introduce phenotypic variation rapidly in response to a stimulus versus obligately sexual reproducers. They note that *Daphnia* species are well known for their phenotypic plasticity, especially in defensive modifications to the exoskeleton. The ‘resetting’ of CpG methylation in mammals for example could prevent such a mechanism of phenotypic plasticity propagating [73]. However, the prevalence of this meiotic reset does vary among vertebrate taxa [73–75], suggesting the potential exists for propagation of DNA methylation changes through sexual reproduction. The mode of methylation in the *Daphnia* genome: low global levels of methylation interspersed with highly methylated gene bodies, is in keeping with sexually reproducing invertebrates [23, 25].

## Conclusions

We have shown that caloric restriction effects the methylation status of a subset of genes in *Daphnia magna*, despite the low overall CpG methylation found in this species. This is in line with recent work into other environmental stressors of *Daphnia*. Functional enrichment analysis of the annotation of loci with changed methylation status identified hypomethylation in the CR treatment of genes involved in methylation and acyl-coenzyme A dehydrogenases as the functionally enriched mode of the response. While we have focused on the effect of caloric restriction on DNA methylation status, there are alternative potential epigenetic responses to CR, including differential expression of small RNAs (sRNAs) and histone modifications. Previously, we established that CR induces differential micro RNA (miRNA) expression in *D. magna* under an equivalent experimental design [48], but other sRNAs, for example piwi interacting RNAs (piRNAs) and transfer RNA-derived small RNA (tsRNA), could also have a role in CR-dependent gene regulation [76, 77]. Histone modification in response to CR or protein restriction are proposed to increase longevity [78] by delaying and repressing ageing-related processes and diseases. Future studies using the tractable *D. magna* model could vary a range of dietary components (overall calories, proteins or fatty acids), and examine the joint effects of a range of epigenetic mechanisms.

## Methods

### *Daphnia magna* preparation and experiment

We used a single clone (known to us as Clone 32) of *D. magna*, collected from the Kaimes pond near Leitholm in the Scottish Borders [79]. Six replicates of control (i.e. well-fed, or normal food (NF)) *D. magna* were compared to six replicates of caloric restricted (CR) *D. magna* to identify differentially methylated regions. Maternal lines were first acclimatized for three generations. For this, individuals were kept in artificial pond medium [80] at 20 °C and on a 12 h:12 h light:dark cycle and fed 2.5 × 10^6^ cells of the single-celled green algae *Chlorella vulgaris* daily. Following three generations of acclimatisation (detailed in [48]), 40 offspring from each mother were isolated and split to form a replicate. Twenty were fed a normal diet of 5 × 10^6^ algal cells/day and the remaining 20 that were fed a CR diet of 1 × 10^6^ algal cells/day. Each replicate was split and reared in four sub-replicate jars of five animals. These were pooled for DNA extraction. Hence, NF and CR replicates were paired by mother and each consisted of 20 individuals in total. The experiment was ended after the birth of second clutch (approximately day 12 of the treatment generation). *D. magna* were ground by motorized pestle in Digsol and proteinase K and incubated overnight at 37 °C and stored at − 70 °C until DNA extraction.

### DNA extraction and sequencing

DNA was extracted from pooled *D. magna* per replicate by phenol-chloroform followed by a Riboshredder RNA digestion step and repeat of the phenol-chloroform step. DNA was eluted into 100 μl of TE buffer and quantified by Qubit fluorimeter Sample purity was checked by 260:280 ratio on Nanodrop, and DNA integrity was examined by running approximately 35 ng DNA on a 0.8% agarose gel stained with ethidium bromide. Each DNA extraction was split in two for creation of a bisulphite converted library and corresponding bisulphite unconverted library (all steps the same except bisulphite conversion). Twenty-four libraries were created: 12 bisulphite-converted and 12 corresponding unconverted samples. This was done to identify per replicate mismatches from the reference and remove false positive methylation calls. All libraries were created by Edinburgh Genomics using the Zymogen EZ DNA Methylation-Lightning Kit and Methylseq Library prep Illumina TruSeq DNA Methylation Kit and 125 base pair paired-end sequenced on Illumina HiSeq. Raw read data have been deposited in the European Nucleotide Archive under accession PRJEB24784 (file names and conversion status, Additional file 6).

### Quality assessment and mapping

Before aligning reads to the *D. magna* reference genome (version 2.4 downloaded from: http://arthropods.eugenes.org/EvidentialGene/daphnia/daphnia_magna/Genes/function/cddrpsdapmaevg14.gotab2), the reference was edited to match the haplotypes present in clone 32 [48]. This conversion increased read mapping efficiency by reducing polymorphism between the reference (assembled from a different clone) and our data.

Reads from both bisulphite converted and unconverted libraries were trimmed of the first and last nine bases of every read using TrimGalore! (version 0.4.1) [81] after initial inspection of Bismark m-bias plots. TrimGalore! was also used to remove base calls with a Phred score of 20 or lower, adapter sequences, and sequences shorter than 20 bases. FastQC 0.11.4 (downloaded from: http://www.bioinformatics.babraham.ac.uk/projects/fastqc) was used to inspect the data. Bisulphite calls were made with Bismark 0.16.3 [59]. Bismark alignments were performed with option “–score_min L,0,-0.6”. PCR duplicates were removed using the *deduplicate_bismark* script. Bismark reports indicated that libraries were not fully bisulphite converted and raw methylation calls were approximately 3% for CpG, CHH and CHG sites. Previous research has shown that CHH and CHG methylation is negligible in the *D. magna* genome [29]. As a result, we used the *filter_non_conversion* script to remove reads that contained CHH and/or CHG methylation sites as indicative of a non-bisulphite converted read. Finally, methylated sites were identified using *bismark_methylation_extractor* and reports created with *bismark2report*.

Further variants were predicted per replicate using the unconverted library reads by following the GATK pipeline [82] and converted strain 32 reference sequence. One round of base quality score recalibration was sufficient using variants previously identified from strain 32. Variants were hard-filtered in GATK using recommendations for single nucleotide polymorphisms (SNPs): quality by depth (QD) > 2, fisher strand (FS) > 60, root mean square of mapping quality (MQ) < 40, mapping quality rank sum test (MQRankSum) < − 12.5 and read position rank sum (ReadPosRankSum) < − 8.0. Sites with SNPs at methylated positions were removed from the analysis using BEDtools [83].

### Differential methylation analysis and region validation

All analyses were performed using methylation calls from the bisulphite converted libraries only. Hierarchical sample clustering of genome-wide methylation patterns across replicates was generated using methylKit [84] and pvclust [85]. A matrix was prepared by adapting R code of the “clusterSamples” command in methyKit and used as input to Pvclust (Additional file 7). The dendrogram was then inferred by hierarchical clustering of correlation-based distances using the Ward.D2 method, confidence in relationships between replicates was provided by 10,000 bootstraps. The 6 NF and six CR replicates were then compared using the bsseq [61] Bioconductor package in R to identify regions of differential methylation, the input files were the same as for hierarchical clustering (Additional file 7). Bsseq uses the Bsmooth algorithm to identify local methylation estimates from low-coverage data, it exploits highly-correlated methylation levels across genomes. *D. magna* is no exception despite low-levels of CpG methylation. It has a “mosaic-like” distribution of methylation as shown by [25]. Regions of differential methylation are then assessed using the biological variation provided by the replicates. We ran bsseq with a paired t-test to incorporate differences between pairs due to mother on methylation. CpG sites were filtered so that at least two replicates in each treatment had six or more reads. We converted locally corrected t-test statistics into *p*-values (with five degrees of freedom, as six replicate pairs minus one) and a significance threshold of 0.05 was applied. *P*-values were converted to q-values using the Bioconductor package “qvalue” [62] and a false discovery rate threshold of 0.05 was applied to each CpG site (p- and q-value R scripts: Additional file 7). Bsseq identified DMRs were selected using a t-statistic cutoff of − 4.6 and 4.6, a greater than 0.1 average difference in methylation between groups, and at least three methylated CpGs. Genes overlapping DMRs were identified using the *D. magna* assembly version 2.4 genome annotation file and BEDTools. This list of overlapping genes was used as a basis for functional enrichment analysis.

To validate bsseq filtered DMR regions we adapted the approach of Herb et al. [20]. We took raw *p*-values per methylated CpG calculated in bsseq, as opposed to bumphunter in [20], and overlapped them with all of the bsseq predicted DMR regions, not the filtered set. These *p*-values were then combined using comb-p [86] to identify shared peaks between the bsseq DMR and comb-p approach. Comb-p is a five-step process that first (1) calculates the correlation between proximal *p*-values of varying distances (the autocorrelation), (2) combines adjacent *p*-values using the Stouffer-Liptak-Kechris correction which incorporates autocorreclation, (3) adjusts for false discovery by the Benjamini-Hochberg correction, (4) identifies enrichment regions as series of low *p*-values, and (5) *p*-value per region is assigned using the Stouffer-Liptak correction. The results of comb-p and bsseq filtered DMRs were intersected and those regions with corrected *p*-values from comb-p were further considered.

### Functional enrichment analysis

Enriched GO (gene ontology) terms were identified using topGO. The weight01 algorithm was applied as it considers the GO hierarchy, a Fisher’s exact test with an α of 0.01 was used to identify enriched genes, and no *p*-value correction was applied as per topGO author recommendation (R code, Additional file 7). Hypo- and hypermethylated genes of interest were tested against databases of GO terms for all *D. magna* genes, those with at least one methylated CpG, and those with at least three CpGs to match the bsseq filtering of DMRs. The per-CpG raw methylation (pre-smoothing) rates were extracted from the Bsmooth object using getMeth in bsseq. Molecular function (MF) and biological process (BP) GO terms are reported. *D. magna* GO terms were downloaded from http://arthropods.eugenes.org/EvidentialGene/daphnia/daphnia_magna/Genes/function/cddrpsdapmaevg14.gotab2.

## Additional files

Additional file 1: Reads sequenced per replicate for both converted and unconverted libraries. Alignments analysed is number of read-pairs per bisulphite converted library aligned by Bismark; deduplicated is number of read pairs after removal of PCR duplicates; non CpG filtered is read-pairs remaining after removal of CHH and CHG containing reads; bases remaining are number of bases left for CpG methylation prediction in Bismark; average coverage is for filtered bases at a *D. magna* genome size of 240 megabases; CpG sites remaining before and after filtering of polymorphic sites, polymorphic sites were identified from unconverted libraries against a reference genome converted to Clone 32; Non CpG % methylated are CHH and CHG sites methylated (100 minus this figure is equivalent to bisulphite conversion efficiency), CpG % methylated is the genome-wide percentage of CpG methylation, and CpG % methylated filtered is the percentage remaining after reads containing CHH or CHG methylation are removed from the analysis. Diet, H: normal food, L: caloric restriction, code: combination of diet and mother. (XLSX 48 kb)

Additional file 2: MultiQC report in html format reporting general statistics from the Bismark alignment process for all replicates. Including alignment rates, deduplication effect, overall cytosine methylation and m-bias plot. This plot shows average methylation level per position across reads, demonstrating minimal bias at 5′ and 3′ reads after trimming of first and last nine base pairs of each read. (HTML 1089 kb)

Additional file 3: Gene annotations for genes with overlapping significantly hypomethylated regions under caloric restriction. Genes overlapped by a DMR of greater than 20% mean difference in methylation level are indicated in column 3. (XLSX 14 kb)

Additional file 4: Gene annotations for genes with overlapping significantly hypermethylated regions under caloric restriction. Genes overlapped by a DMR of greater than 20% mean difference in methylation level are indicated in column 3. (XLSX 12 kb)

Additional file 5: GO term hierarchies for the two enriched GO terms: A) Biological Process and B) Molecular Function. Ovals and rectangles contain GO identification, GO name, *p*-value and number of genes tested and over total number of genes from that category. Red rectangles represent significant terms under the topGO criteria applied. (PS 91 kb)

Additional file 6: Raw read files used in this study deposited in the European Nucleotide Archive under accession PRJEB24784. Treatment, H: normal food and L: caloric restricted. (XLSX 34 kb)

Additional file 7: R code used in methylKit, pvclust, bsseq, and comb-p sections of this study. (TXT 8 kb)

## Abbreviations

ABCD1: ATP-binding cassette sub-family D member 1
AMP: Adenosine monophosphate
ATP: Adenosine triphosphate
bp: Base pairs
BP: Biological process
CAM-KK: Calcium/calmodulin-dependent protein kinase kinases
CHH/CHG: Cytosine followed by nucleotides where H is adenine, thymine or cytosine and G is guanine
CpG: Cytosine followed by guanine
CR: Caloric restriction
DMR: Differentially methylated region
DNA: Deoxyribonucleic acid
DNMT: DNA methyltransferase
ENA: European Nucleotide Archive
FDR: False discovery rate
GMP: Guanosine monophosphate
GO: Gene ontology
IAP: Intracisternal A particle
MCM2: Minichromosome maintenance complex component 2
MF: Molecular function
miRNA: micro RNA
NAD: Nicotinamide adenine dinucleotide
NF: Normal food
PCR: Polymerase chain reaction
piRNA: piwi interacting RNA
RNA: Ribonucleic acid
SMC2: Structural maintenance of chromosomes protein 2
sRNA: small RNA
TE: Tris (hydroxymethyl) aminomethane ethylenediaminetetraacetic acid
tsRNA: transfer RNA-derived small RNA
WGBS: Whole genome bisulphite sequencing

## References

[1] Egger G, Liang G, Aparicio A, Jones PA (2004). Epigenetics in human disease and prospects for epigenetic therapy. Nature.

[2] Cooper TM, Mockett RJ, Sohal BH, Sohal RS, Orr WC (2004). Effect of caloric restriction on life span of the housefly, *Musca domestica*. FASEB J FASEB.

[3] Forster MJ, Morris P, Sohal RS (2003). Genotype and age influence the effect of caloric intake on mortality in mice. FASEB J FASEB.

[4] Colman RJ, Anderson RM, Johnson SC, Kastman EK, Kosmatka KJ, Beasley TM (2009). Caloric restriction delays disease onset and mortality in rhesus monkeys. Science (80-. ).

[5] Weindruch R (1996). The retardation of aging by caloric restriction: studies in rodents and primates. Toxicol. Pathol.

[6] Weindruch R, Walford RL. Retardation of aging and disease by dietary restriction. Springfield: CC Thomas; 1988.

[7] Saiz E, Calbet A, Griffell K, Bersano JGF, Isari S, Solé M (2015). Ageing and caloric restriction in a marine planktonic copepod. Sci Rep.

[8] Xiang H, Zhu J, Chen Q, Dai F, Li X, Li M (2010). Single base-resolution methylome of the silkworm reveals a sparse epigenomic map. Nat Biotechnol.

[9] Clark J, Garbutt JS, McNally L, Little TJ (2017). Disease spread in age structured populations with maternal age effects. Ecol Lett Wiley Online Library.

[10] Garbutt JS, Little TJ (2014). Maternal food quantity affects offspring feeding rate in *Daphnia magna*. Biol. Lett.

[11] Kirkwood TBL, Shanley DP (2005). Food restriction, evolution and ageing. Mech. Ageing Dev. Elsevier.

[12] Sohal RS, Weindruch R (1996). Oxidative Stress, Caloric Restriction, and Aging. Science (80-. ).

[13] Fernandes G, Yunis EJ, Good RA (1976). Suppression of adenocarcinoma by the immunological consequences of calorie restriction. Nature.

[14] Sarkar NH, Fernandes G, Telang NT, Kourides IA, Good RA (1982). Low-calorie diet prevents the development of mammary tumors in C3H mice and reduces circulating prolactin level, murine mammary tumor virus expression, and proliferation of mammary alveolar cells. Proc. Natl. Acad. Sci. National Acad Sciences.

[15] Kubo C, Johnson BC, Good RA. A crucial influence of total calorie intake on autoimmune-prone mice: influence of diets of grossly different composition on immunologic functions. Fed Proc. 1984;3:2249.

[16] Duan W, Mattson MP (1999). Dietary restriction and 2-deoxyglucose administration improve behavioral outcome and reduce degeneration of dopaminergic neurons in models of Parkinson’s disease. J Neurosci Res Wiley Online Library.

[17] Zhu H, Guo Q, Mattson MP (1999). Dietary restriction protects hippocampal neurons against the death-promoting action of a presenilin-1 mutation. Brain Res Elsevier.

[18] Bewick AJ, Vogel KJ, Moore AJ, Schmitz RJ (2017). Evolution of DNA methylation across insects. Mol Biol Evol.

[19] Cardoso-Júnior CAM, Guidugli-Lazzarini KR, Hartfelder K (2018). DNA methylation affects the lifespan of honey bee (*Apis mellifera L.*) workers-evidence for a regulatory module that involves vitellogenin expression but is independent of juvenile hormone function. Insect Biochem. Mol. Biol. Elsevier.

[20] Herb BR, Shook MS, Fields CJ, Robinson GE (2018). Defense against territorial intrusion is associated with DNA methylation changes in the honey bee brain. BMC Genomics.

[21] Asselman J, De Coninck DIM, Vandegehuchte MB, Jansen M, Decaestecker E, De Meester L (2015). Global cytosine methylation in *Daphnia magna* depends on genotype, environment, and their interaction. Environ Toxicol Chem Wiley Online Library.

[22] Asselman J, De Coninck DIM, Beert E, Janssen CR, Orsini L, Pfrender ME (2017). Bisulfite sequencing with Daphnia highlights a role for epigenetics in regulating stress response to *Microcystis* through preferential differential methylation of serine and threonine amino acids. Environ. Sci. Technol..

[23] Jeremias G, Barbosa J, Marques SM, De Schamphelaere KAC, Van Nieuwerburgh F, Deforce D (2018). Transgenerational inheritance of DNA Hypomethylation in *Daphnia magna* in response to salinity stress. Environ. Sci. Technol..

[24] Trijau M, Asselman J, Armant O, Adam-Guillermin C, De Schamphelaere KAC, Alonzo F (2018). Transgenerational DNA methylation changes in *Daphnia magna* exposed to chronic γ irradiation. Environ Sci Technol.

[25] Kvist J, Gonçalves Athanàsio C, Shams Solari O, Brown JB, Colbourne JK, Pfrender ME (2018). Pattern of DNA methylation in *Daphnia*: evolutionary perspective. Genome Biol. Evol..

[26] Bestor TH (1992). Activation of mammalian DNA methyltransferase by cleavage of a Zn binding regulatory domain. EMBO J.

[27] Ehrlich M, Gama-Sosa MA, Huang L-H, Midgett RM, Kuo KC, McCune RA (1982). Amount and distribution of 5-methylcytosine in human DNA from different types of tissues or cells. Nucleic Acids Res.

[28] Olson CE, Roberts SB (2014). Genome-wide profiling of DNA methylation and gene expression in *Crassostrea gigas* male gametes. Front Physiol.

[29] Asselman J, De Coninck DIM, Pfrender ME, De Schamphelaere KAC (2016). Gene body methylation patterns in *Daphnia* are associated with gene family size. Genome Biol Evol.

[30] Zemach A, IE MD, Silva P, Zilberman D (2010). Genome-wide evolutionary analysis of eukaryotic DNA methylation. Science (80-. ).

[31] Sarda S, Zeng J, Hunt BG, Yi SV (2012). The evolution of invertebrate gene body methylation. Mol. Biol. Evol.

[32] Bonasio R, Li Q, Lian J, Mutti NS, Jin L, Zhao H (2012). Genome-wide and caste-specific DNA methylomes of the ants *Camponotus floridanus* and *Harpegnathos saltator*. Curr Biol Elsevier.

[33] Suzuki MM, Kerr ARW, De Sousa D, Bird A (2007). CpG methylation is targeted to transcription units in an invertebrate genome. Genome Res.

[34] Flores K, Wolschin F, Corneveaux JJ, Allen AN, Huentelman MJ, Amdam GV (2012). Genome-wide association between DNA methylation and alternative splicing in an invertebrate. BMC Genomics.

[35] Li-Byarlay H, Li Y, Stroud H, Feng S, Newman TC, Kaneda M (2013). RNA interference knockdown of DNA methyl-transferase 3 affects gene alternative splicing in the honey bee. Proc Natl Acad Sci National Acad Sciences.

[36] Li S, Zhang J, Huang S, He X (2017). Genome-wide analysis reveals that exon methylation facilitates its selective usage in the human transcriptome. Brief Bioinform.

[37] Horvath S (2013). DNA methylation age of human tissues and cell types. Genome Biol.

[38] Horvath S (2015). Erratum to: DNA methylation age of human tissues and cell types. Genome Biol.

[39] Pal S, Tyler JK (2016). Epigenetics and aging. Sci Adv.

[40] Jung M, Pfeifer GP. Aging and DNA methylation. BMC Biol. BioMed Central. 2015;13:–7. 10.1186/s12915-015-0118-4 PMC4311512 25637097

[41] Li Y, Liu L, Tollefsbol TO (2010). Glucose restriction can extend normal cell lifespan and impair precancerous cell growth through epigenetic control of hTERT and p16 expression. FASEB J. FASEB.

[42] Hahn O, Grönke S, Stubbs TM, Ficz G, Hendrich O, Krueger F (2017). Dietary restriction protects from age-associated DNA methylation and induces epigenetic reprogramming of lipid metabolism. Genome Biol.

[43] Lane MA, Tilmont EM, De Angelis H, Handy A, Ingram DK, Kemnitz JW (2000). Short-term calorie restriction improves disease-related markers in older male rhesus monkeys (*Macaca mulatta*). Mech Ageing Dev Elsevier.

[44] Kim CH, Lee EK, Choi YJ, An HJ, Jeong HO, Park D (2016). Short-term calorie restriction ameliorates genomewide, age-related alterations in DNA methylation. Aging Cell.

[45] Latta LC, Frederick S, Pfrender ME (2011). Diet restriction and life history trade-offs in short-and long-lived species of *Daphnia*. J. Exp. Zool. Part a Ecol. Genet. Physiol.

[46] Garbutt JS, Little TJ (2017). Bigger is better: changes in body size explain a maternal effect of food on offspring disease resistance. Ecol Evol.

[47] Coakley CM, Nestoros E, Little TJ (2018). Testing hypotheses for maternal effects in *Daphnia magna*. J Evol Biol.

[48] Hearn J, Chow FW-N, Barton H, Tung M, Wilson P, Blaxter M (2018). *Daphnia magna* microRNAs respond to nutritional stress and ageing but are not transgenerational. Mol Ecol.

[49] Pietrzak B (2011). Interclonal differences in age-specific performance in *Daphnia magna*. J Limnol.

[50] Lynch M (1989). The life history consequences of resource depression in *Daphnia pulex*. Ecology Wiley Online Library.

[51] Dudycha JL (2003). A multi-environment comparison of senescence between sister species of *Daphnia*. Oecologia Springer.

[52] McCauley E, Murdoch WW, Nisbet RM. Growth, reproduction, and mortality of *Daphnia pulex Leydig*: life at low food. Funct Ecol JSTOR. 1990;4:505–14.

[53] Schwartz TS, Pearson P, Dawson J, Allison DB, Gohlke JM (2016). Effects of fluctuating temperature and food availability on reproduction and lifespan. Exp Gerontol Elsevier.

[54] Kim E, Ansell CM, Dudycha JL (2014). Resveratrol and food effects on lifespan and reproduction in the model crustacean *Daphnia*. J. Exp. Zool. A. Ecol. Genet. Physiol.

[55] Anderson BG (1932). The number of pre-adult instars, growth, relative growth, and variation in *Daphnia magna*. Biol Bull.

[56] Agar WE (1930). A statistical study of regeneration in two species of Crustacea. J Exp Biol.

[57] Vandegehuchte MB, De Coninck D, Vandenbrouck T, De Coen WM, Janssen CR (2010). Gene transcription profiles, global DNA methylation and potential transgenerational epigenetic effects related to Zn exposure history in *Daphnia magna*. Environ Pollut Elsevier.

[58] Vandegehuchte MB, Kyndt T, Vanholme B, Haegeman A, Gheysen G, Janssen CR (2009). Occurrence of DNA methylation in *Daphnia magna* and influence of multigeneration cd exposure. Environ Int Elsevier.

[59] Krueger F, Andrews SR (2011). Bismark: a flexible aligner and methylation caller for bisulfite-Seq applications. Bioinformatics. Oxford University Press.

[60] Porter J, Sun MAN, Xie H, Zhang L (2015). Investigating bisulfite short-read mapping failure with hairpin bisulfite sequencing data. BMC Genomics.

[61] Hansen KD, Langmead B, Irizarry RA (2012). BSmooth: from whole genome bisulfite sequencing reads to differentially methylated regions. Genome Biol.

[62] Storey JD, Tibshirani R (2003). Statistical significance for genomewide studies. Proc. Natl. Acad. Sci.

[63] Orsini L, Gilbert D, Podicheti R, Jansen M, Brown JB, Solari OS (2016). *Daphnia magna* transcriptome by RNA-Seq across 12 environmental stressors. Sci. Data.

[64] Hunt BG, Glastad KM, Yi SV, Goodisman MAD (2013). The function of intragenic DNA methylation: insights from insect epigenomes. Integr. Comp. Biol..

[65] Blanco S, Frye M (2014). Role of RNA methyltransferases in tissue renewal and pathology. Curr Opin Cell Biol Elsevier.

[66] Schosserer M, Minois N, Angerer TB, Amring M, Dellago H, Harreither E (2015). Methylation of ribosomal RNA by NSUN5 is a conserved mechanism modulating organismal lifespan. Nat. Commun.

[67] Züst R, Cervantes-Barragan L, Habjan M, Maier R, Neuman BW, Ziebuhr J (2011). Ribose 2′-O-methylation provides a molecular signature for the distinction of self and non-self mRNA dependent on the RNA sensor Mda5. Nat. Immunol.

[68] Daffis S, Szretter KJ, Schriewer J, Li J, Youn S, Errett J (2010). 2′-O methylation of the viral mRNA cap evades host restriction by IFIT family members. Nature.

[69] Matern D, Rinaldo P, Adam M, Ardinger H, Pagon R (1993). Medium-Chain Acyl-Coenzyme A Dehydrogenase Deficiency.

[70] Ma DK, Li Z, Lu AY, Sun F, Chen S, Rothe M (2015). Acyl-CoA dehydrogenase drives heat adaptation by sequestering fatty acids. Cell. Elsevier.

[71] Katewa SD, Demontis F, Kolipinski M, Hubbard A, Gill MS, Perrimon N (2012). Intramyocellular fatty-acid metabolism plays a critical role in mediating responses to dietary restriction in *Drosophila melanogaster*. Cell Metab Elsevier.

[72] Harris KDM, Bartlett NJ, Lloyd VK. *Daphnia* as an emerging epigenetic model organism. Genet Res Int Hindawi. 2012;2012:147892:1–147892:8. 10.1155/2012/147892 PMC3335723 22567376

[73] Seisenberger S, Peat JR, Hore TA, Santos F, Dean W, Reik W (2013). Reprogramming DNA methylation in the mammalian life cycle: building and breaking epigenetic barriers. Phil. Trans. R. Soc. B.

[74] Potok ME, Nix DA, Parnell TJ, Cairns BR (2013). Reprogramming the maternal zebrafish genome after fertilization to match the paternal methylation pattern. Cell. Elsevier.

[75] Wang L, Zhang J, Duan J, Gao X, Zhu W, Lu X (2014). Programming and inheritance of parental DNA methylomes in mammals. Cell Elsevier.

[76] Chen Q, Shi J, Peng H, Zhang X, Zhang Y, Qian J (2016). Sperm tsRNAs contribute to intergenerational inheritance of an acquired metabolic disorder. Science (80-. ).

[77] Brennecke J, Malone CD, Aravin AA, Sachidanandam R, Stark A, Hannon GJ (2008). An epigenetic role for maternally inherited piRNAs in transposon silencing. Science (80-. ).

[78] Li Y, Daniel M, Tollefsbol TO (2011). Epigenetic regulation of caloric restriction in aging. BMC Med.

[79] Auld SKJR, Hall SR, Housley Ochs J, Sebastian M, Duffy MA (2014). Predators and patterns of within-host growth can mediate both among-host competition and evolution of transmission potential of parasites. Am. Nat.

[80] Klüttgen B, Dülmer U, Engels M, Ratte HT (1994). ADaM, an artificial freshwater for the culture of zooplankton. Water Res Elsevier.

[81] Krueger F. Trim galore. A wrapper tool around Cutadapt FastQC to consistently apply Qual. Adapt. trimming to FastQ files. 2015.

[82] Van Der Auwera GA, Carneiro MO, Hartl C, Poplin R, Levy-Moonshine A, Jordan T, et al. From FastQ data to high confidence varant calls: the genome analysis toolkit best practices pipeline. Curr Protoc Bioinforma. 2014;11:11.10.1–11.10.33. 10.1002/0471250953.bi1110s43 PMC4243306 25431634

[83] Quinlan AR, Hall IM (2010). BEDTools: a flexible suite of utilities for comparing genomic features. Bioinformatics..

[84] Akalin A, Kormaksson M, Li S, Garrett-Bakelman FE, Figueroa ME, Melnick A (2012). methylKit: a comprehensive R package for the analysis of genome-wide DNA methylation profiles. Genome Biol.

[85] Suzuki R, Shimodaira H (2006). Pvclust: an R package for assessing the uncertainty in hierarchical clustering. Bioinformatics.

[86] Pedersen BS, Schwartz DA, Yang IV, Kechris KJ. Comb-p : software for combining, analyzing, grouping and correcting spatially correlated *P* -values. Bioinformatics. 2012;28:2986–8. 10.1093/bioinformatics/bts545 PMC3496335 22954632

